# Combined loss of histone methyltransferases GLP and G9a disrupts neural crest cell fate determination

**DOI:** 10.1172/JCI205970

**Published:** 2026-08-13

**Authors:** Diana B. Voss, Emily J. Shields, Andrey Poleshko, Li Li, Jun Li, Lizhou Wang, Mariacristina Calcagno, Rajan Jain, Cheryl L. Smith, Jonathan A. Epstein.

**Affiliations:** 1 Department of Cell and Developmental Biology and; 2 Department of Medicine, Perelman School of Medicine, University of Pennsylvania, Philadelphia, Pennsylvania, USA.

**Keywords:** Bone biology, Cardiology, Development, Epigenetics

## Abstract

Mice missing two chromatin-regulating enzymes in developing face and heart cells show cleft palate and heart defects, mirroring a human genetic disorder.

## To the Editor:

Mutations in *EHMT1*, the gene encoding the histone methyltransferase GLP, are associated with Kleefstra syndrome, a rare multisystemic disorder with craniofacial and cardiovascular abnormalities (1). The mechanisms by which the loss of histone methyltransferase could lead to Kleefstra syndrome remain poorly understood. *EHMT1*, and its paralog *EHMT2*, which encodes G9a, catalyze the methylation of lysine 9 on histone H3 (H3K9) (2). Given the importance of neural crest to craniofacial and cardiovascular morphogenesis, as well as the link between GLP mutations and anatomical defects in these neural crest–derived tissues, we sought to assess the impact of histone methyltransferase loss on neural crest development. We crossed mice in which critical exons of *Ehmt1* and *Ehmt2* were floxed with mice expressing Cre recombinase in premigratory neural crest via *Wnt1*-*Cre* (3). Additionally, *Rosa26^TdTomato/+^* was used to lineage trace neural crest–derived cells (NCCs; Supplemental Figure 1A; supplemental material available online with this article; https://doi.org/10.1172/JCI205970DS1).

Expression of GLP and G9a was attenuated at E10.5 (Supplemental Figure 1B) and undetectable at E11.5, specifically in NCC-derived pharyngeal mesenchyme in GLP/G9a–double knockout (DKO; *Ehmt1^fl/fl^ Ehmt2^fl/fl^ Rosa^TdTomato/+^ Wnt1-Cre^+^*) embryos compared with control (Figure 1A). Additionally, RNAscope showed reduction of *Ehmt1* and *Ehmt2* mRNA (Supplemental Figure 1C). We observed markedly reduced H3K9me2 but stable H3K9me1 and H3K9me3 levels in NCCs populating the branchial arches and outflow tract in DKO E11.5 embryos (Figure 1A and Supplemental Figure 1D). Isolated NCCs from DKO and control embryos showed persistent reduction of H3K9me2 at E13.5 (Supplemental Figure 1, E and F). Thus, GLP and G9a loss in NCCs reduces H3K9me2 but not H3K9me1 or H3K9me3 in vivo. Antibody specificity was confirmed in a peptide competition assay (Supplemental Figure 1G).

Having established GLP and G9a deletion, we investigated their role in NCC morphogenesis. DKO embryos were born at normal Mendelian ratios, but many died perinatally and by P14, no surviving DKO pups were identified (0 of 30; Supplemental Figure 2A). On P0, DKO pups, unlike controls, showed microcephaly and lacked a milk spot, suggesting they could not suckle (Supplemental Figure 2, B and C). All DKO pups also exhibited cleft palates as well as hypoplasia of NCC-derived skull bones (Figure 1B and Supplemental Figure 2, D–G). There were, however, no differences in the number of ribs or tibia lengths compared with controls (Supplemental Figure 2, H–J).

Given the critical role for neural crest in mediating outflow tract septation and remodeling of the aortic arch arteries, we also examined cardiac morphology. We observed instances of patent ductus arteriosus (4 of 5 P0 pups; Figure 1C and Supplemental Figure 2, K–M) and double outlet right ventricle with ventricular septal defects (2 of 3 E17.5 embryos; Supplemental Figure 2N) in DKOs but not in controls.

We sought to understand the basis for phenotypes observed in DKOs. We found no differences in the number of NCCs populating the branchial arches, nor in the percentage of proliferating or apoptotic NCCs in E11.5 DKO embryos compared with controls (Supplemental Figure 3A). NCCs normally generate ectomesenchyme-derived bone (4); however, we observed reduced expression of the osteogenic proteins RUNX2 and SP7 in the oral cavity of DKO embryos compared with controls (Figure 1D and Supplemental Figure 3B). NCCs also give rise to some smooth muscle cells surrounding the aortic arch arteries (5). We observed a marked reduction in NCCs expressing smooth muscle actin surrounding the aortic arch arteries in DKO embryos compared with controls (Figure 1E and Supplemental Figure 3C). These data suggest GLP and G9a regulate differentiation of NCCs during craniofacial and vascular development.

To further characterize the impact of GLP and G9a loss on differentiation, we performed RNA-seq on TdTomato^+^ NCCs isolated from E11.5 branchial arches (Supplemental Figure 4, A–C). In comparisons between control and DKO, we observed no significant expression differences for gene sets associated with proliferation, apoptosis, or migration (Supplemental Figure 4D). However, analysis revealed significant depletion of neural crest and osteoblast lineage gene sets and enrichment for cardiac muscle, endothelial, and myofibroblast lineage gene sets in DKO relative to controls (Figure 1F and Supplemental Figure 4, E–G). Consistent with the expression changes, analysis of CUT&RUN data from control TdTomato^+^ NCCs from E11.5 branchial arches revealed a significantly higher H3K9me2 signal at genes upregulated in DKOs compared with all genes or DKO downregulated genes (Figure 1, G and H). Complementary analysis showed significant enrichment of H3K9me2 at DKO upregulated genes and depletion at downregulated genes relative to random chance (Figure 1I). These results indicate that GLP and G9a loss in NCCs compromises neural crest gene expression and facilitates expression of alternative cell fate pathways. Furthermore, these results suggest that GLP/G9a regulation of H3K9me2 could underlie aspects of craniofacial and cardiovascular defects in some congenital disorders, including Kleefstra syndrome.

For detailed methods, information regarding sex as a biological variable, statistics, supporting data values, study approval, acknowledgments, and author contributions, see the supplemental materials.

## Conflict of interest

The authors have declared that no conflicts of interest exist.

## Funding support

This work is the result of NIH funding, in whole or in part, and is subject to the NIH Public Access Policy. Through acceptance of this federal funding, the NIH has been given the right to make the work publicly available in PubMed Central.

- NIH R35HL140018 (to JAE).
- The Cotswold Foundation (to JAE).
- NIH R35HL166663 (to RJ).
- Burroughs Wellcome Foundation Career Award for Medical Scientists (to RJ).
- W.W. Smith Charitable Trust (to RJ).
- NIH R21 AG081795 (to AP).
- Additional Ventures Catalyst to Independence award (to DBV).
- Abramson Cancer Center National Cancer Institute grant to Penn Cytomics (P30 016520).

## Supplementary Material

Supplemental data

Supporting data values

## Figures and Tables

**Figure 1:**
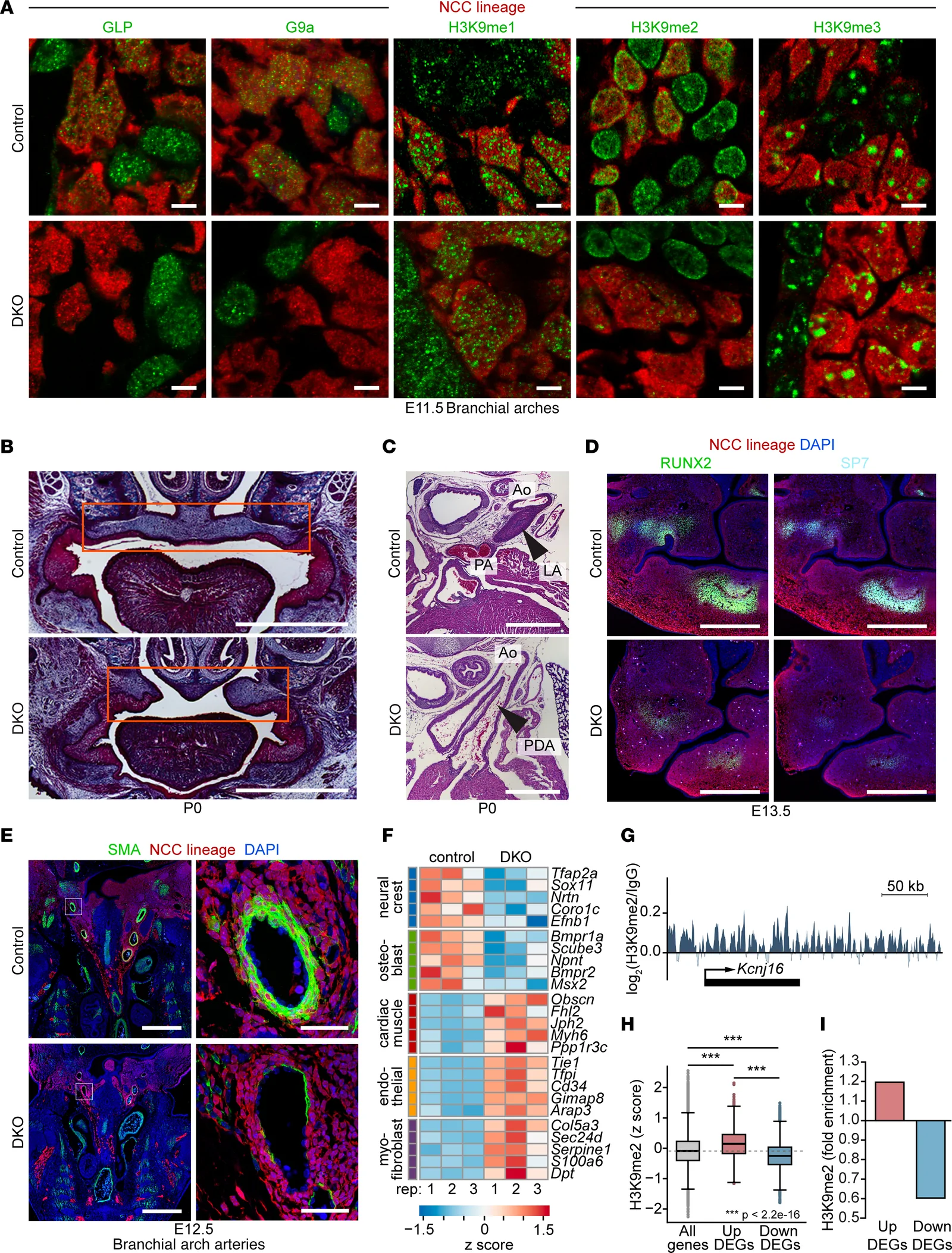
GLP and G9a loss disrupts neural crest differentiation. (**A**) E11.5 control and DKO embryo immunofluorescence (IF) for TdTomato showing NCC lineage (red), costained for GLP, G9a, H3K9me1, H3K9me2, or H3K9me3 (green). Scale bars: 5 μm. (**B**) Masson’s trichrome staining of palatal shelves (boxes) showing cleft palate in P0 DKO embryo. Scale bars: 500 μm. (**C**) H&E staining of P0 transverse sections, with black arrowheads denoting ligamentum arteriosum (LA) in control or patent ductus arteriosus (PDA) in DKO. Ao, aorta; PA, pulmonary artery. Scale bars: 500 μm. (**D**) RUNX2 (green) or SP7 (cyan) and TdTomato (red) IF in oral cavity (DAPI, blue). Scale bars: 500 μm. (**E**) Smooth muscle actin (SMA) (green) and TdTomato (red) IF of branchial arch arteries (DAPI, blue). Scale bars: 500 μm (left), 50 μm (right). (**F**) RNA-seq from E11.5 branchial arch TdTomato^+^ NCCs shown as heatmaps with 5 of the top 10 genes from lineage-specific gene sets differentially enriched in control or DKO. (**G**–**I**) H3K9me2 signal from TdTomato^+^ NCCs. (**G**) H3K9me2-enriched gene locus. (**H**) Distributions across all genes and DKO up- or downregulated genes. Wilcoxon’s rank-sum tests, median of all genes (dashed line). ***P < 2.2 × 10^-16^. Boxes represent 25th–75th percentile range, solid horizontal lines indicate the median, and whiskers are 10th–90th percentiles. (**I**) Fold enrichment at DKO differentially expressed genes (DEGs) using Genomic Association Tester.

## Version 1 08/13/2026

In-Press Preview

## Version 2 10/15/2026

Electronic publication

## Version 3 10/17/2026

Corrected co-corresponding authors note
