# Supplementary material for: Combined loss of histone methyltransferases GLP and G9a disrupts neural crest cell fate determination
Source: J Clin Invest. 2026 Aug 13;136(20):e205970. doi: 10.1172/JCI205970 (PMC13668620; doi:10.1172/JCI205970)
Supplement: Supplementary file 1 [file jci-136-205970-s002.pdf]

Supplemental Figures

S. Fig. 1

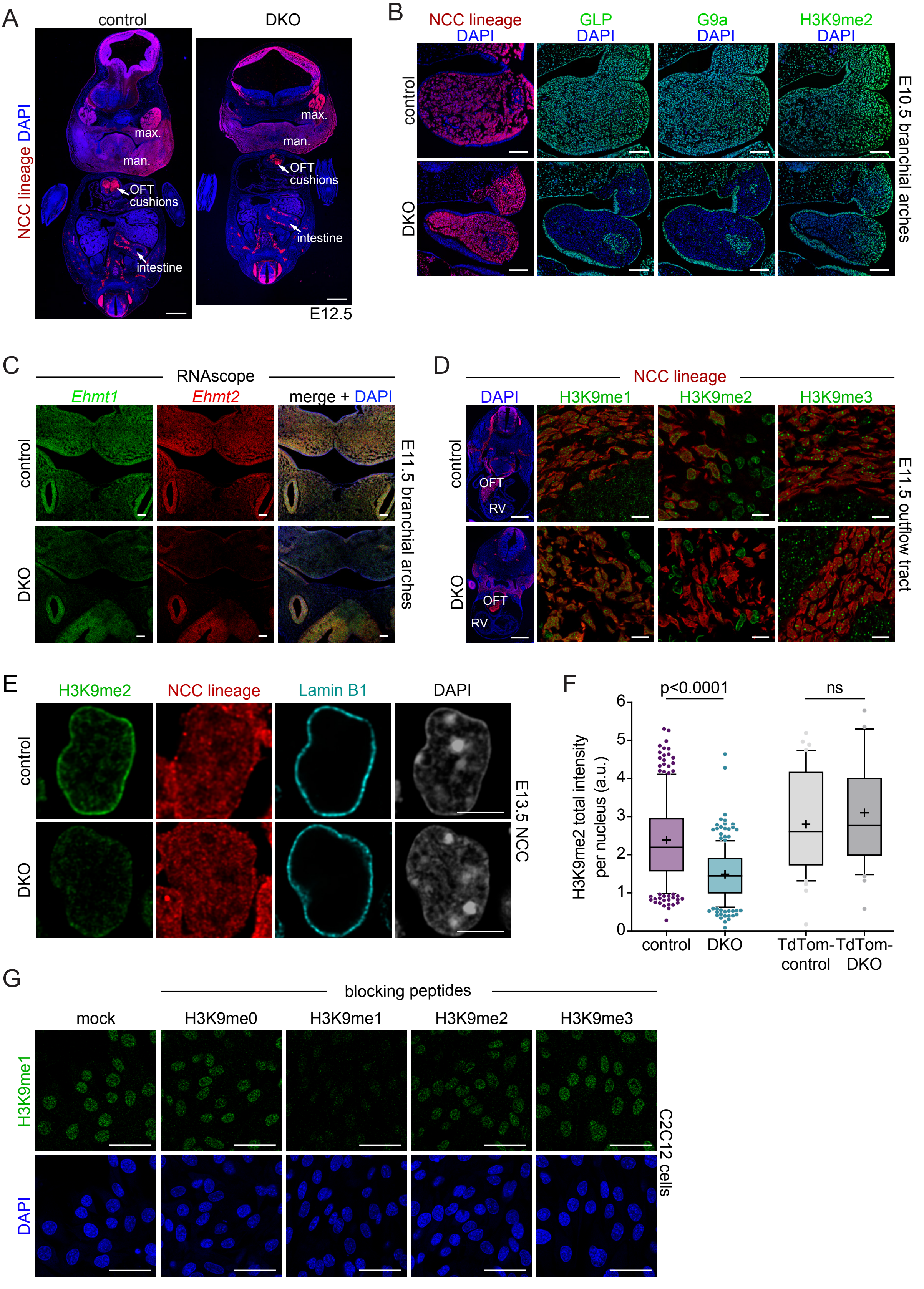

Supplemental Figure 1

(A) Immunofluorescent (IF) images of cross sections through E12.5 control and DKO embryos with DAPI (blue) and TdTomato (NCC lineage, red), scale=500μm. max: maxillary arch, man: mandibular arch, OFT: outflow tract.

(B) IF images at the level of the maxillary arches from E10.5 control and DKO embryos with DAPI (blue), TdTomato (NCC lineage, red), GLP, G9a, or H3K9me2 (green), scale=100μm.

(C) RNAscope of cross sections at the level of the maxillary and mandibular arches from E11.5 control (*Ehmt1$^{f/+}$;Ehmt2$^{f/f}$;Wnt1-Cre-*) and DKO embryos with *Ehmt1* (green), *Ehmt2* (red) and DAPI (blue), scale=500μm.

(D) IF images of cross sections at the level of the cardiac outflow tracts of E11.5 embryos with DAPI (blue), TdTomato (NCC lineage, red), and H3K9 methylation marks as indicated (green), OFT: outflow tract, RV: right ventricle, scale=500μm (left), 25μm (right).

(E) Isolated E13.5 NCCs stained for H3K9me2 (green), TdTomato (red), Lamin B1 (cyan), DAPI (gray), scale=5μm.

(F) Box plots showing total levels of H3K9me2 histone modification in individual nuclei from control and DKO E13.5 NCCs and non-NCCs (TdTomato-negative). The box represents the 25-75th percentile range, median indicated by a horizontal line and mean by "+". Whiskers represent 10th–90th percentiles. n =224, 205, 40, and 31 nuclei per

condition. Statistical analysis was performed using the Mann-Whitney test. ns: not significant.

(G) Representative images of mouse C2C12 cells stained for H3K9me1 (green) and DAPI (blue). Prior to staining, anti-H3K9me1 antibody was pre-incubated with buffer (mock) or one of the indicated blocking peptides, scale=50μm.

S. Fig. 2

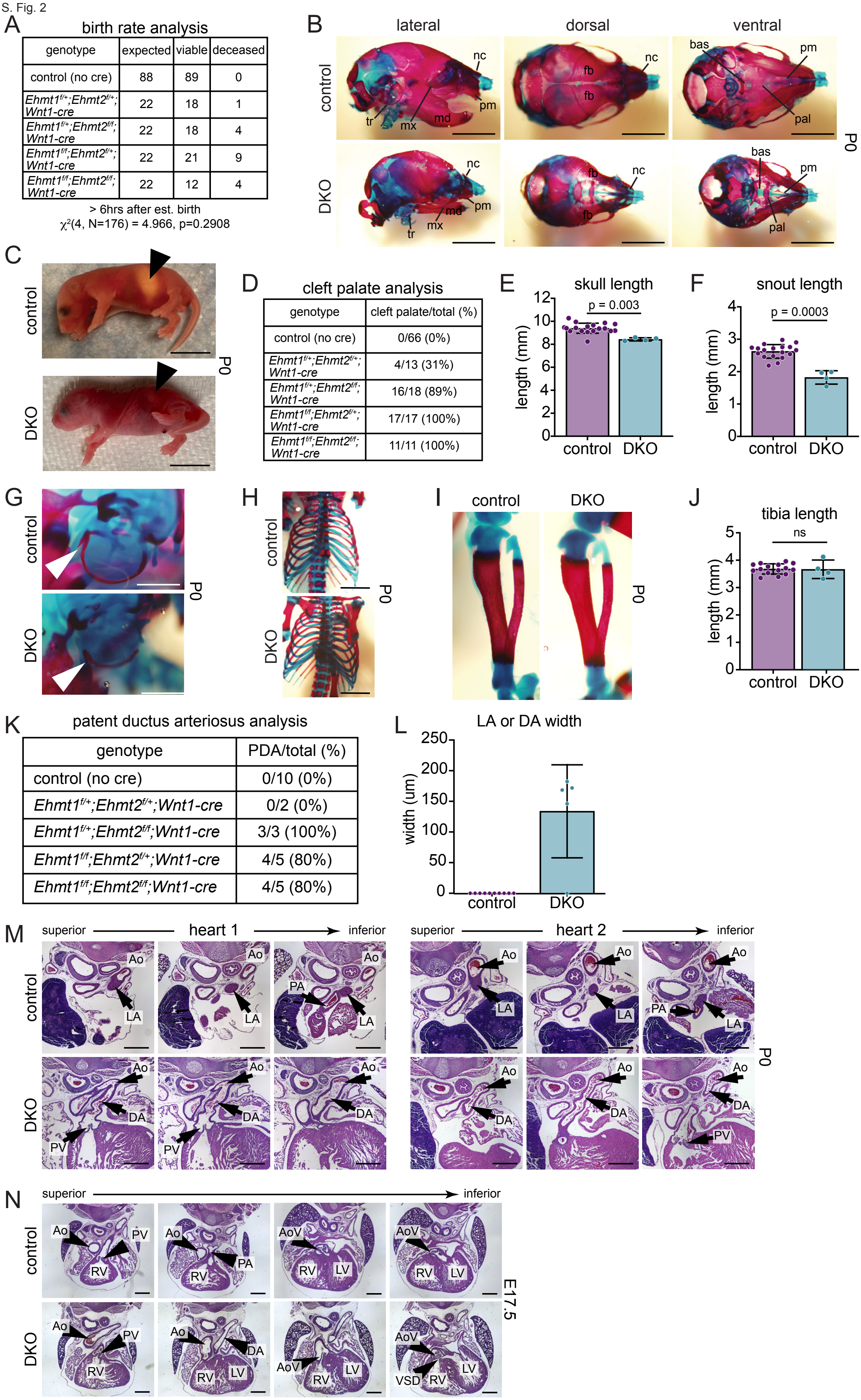
A
birth rate analysis
genotype | expected | viable | deceased
control (no cre) | 88 | 89 | 0
Ehmt1$^{f/+}$;Ehmt2$^{f/+}$;Wnt1-cre | 22 | 18 | 1
Ehmt1$^{f/+}$;Ehmt2$^{f/f}$;Wnt1-cre | 22 | 18 | 4
Ehmt1$^{f/f}$;Ehmt2$^{f/+}$;Wnt1-cre | 22 | 21 | 9
Ehmt1$^{f/f}$;Ehmt2$^{f/f}$;Wnt1-cre | 22 | 12 | 4
> 6hrs after est. birth
$\chi^2(4, N=176) = 4.966, p=0.2908$
B
lateral
dorsal
ventral
control
DKO
nc
pm
md
mx
tr
fb
bas
pal
P0
C
control
DKO
P0
D
cleft palate analysis
genotype | cleft palate/total (%)
control (no cre) | 0/66 (0%)
Ehmt1$^{f/+}$;Ehmt2$^{f/+}$;Wnt1-cre | 4/13 (31%)
Ehmt1$^{f/+}$;Ehmt2$^{f/f}$;Wnt1-cre | 16/18 (89%)
Ehmt1$^{f/f}$;Ehmt2$^{f/+}$;Wnt1-cre | 17/17 (100%)
Ehmt1$^{f/f}$;Ehmt2$^{f/f}$;Wnt1-cre | 11/11 (100%)
E
skull length
p = 0.003
length (mm)
control
DKO
F
snout length
p = 0.0003
length (mm)
control
DKO
G
control
DKO
P0
H
control
DKO
P0
I
control
DKO
P0
J
tibia length
ns
length (mm)
control
DKO
K
patent ductus arteriosus analysis
genotype | PDA/total (%)
control (no cre) | 0/10 (0%)
Ehmt1$^{f/+}$;Ehmt2$^{f/+}$;Wnt1-cre | 0/2 (0%)
Ehmt1$^{f/+}$;Ehmt2$^{f/f}$;Wnt1-cre | 3/3 (100%)
Ehmt1$^{f/f}$;Ehmt2$^{f/+}$;Wnt1-cre | 4/5 (80%)
Ehmt1$^{f/f}$;Ehmt2$^{f/f}$;Wnt1-cre | 4/5 (80%)
L
LA or DA width
width (um)
control
DKO
M
superior
heart 1
inferior
heart 2
control
DKO
Ao
LA
PA
DA
PV
P0
N
superior
inferior
control
DKO
Ao
PV
PA
RV
LV
AoV
DA
VSD
E17.5

Supplemental Figure 2

(A) Table of birth rate and viability at P0 by genotype in the cross between *Ehmt1$^{f/f}$;Ehmt2$^{f/f}$* females with *Ehmt1$^{f/+}$;Ehmt2$^{f/+}$;Wnt1-Cre*+ males. Birth rate calculated from 26 litters of P0 pups (n=176 pups). Chi square calculated based on expected versus observed for each genotype regardless of viability.

(B) Lateral, dorsal, and ventral views of representative skulls from P0 control (*Ehmt1$^{f/f}$;Ehmt2$^{f/f}$;Wnt1-Cre-*) and DKO pups stained with alcian blue (cartilage) and alizarin red (bone). bas: basisphenoid; fb: frontal bone; md: mandible; mx: maxillary; nc: nasal capsule; pal: palatal; pm: premaxillary; tr: tympanic ring; scale=3mm.

(C) P0 pups with black arrow heads on lower left abdomen indicating milk spot in control (*Ehmt1$^{f/+}$;Ehmt2$^{f/f}$;Wnt1-Cre-*) and absence in DKO, scale=1cm.

(D) Table of cleft palate rates observed in animals age E15.5 to P0 by genotype.

(E-F) Quantification of skull lengths (E) and snout lengths (F) for control (*Wnt1-Cre-*) (n=18) and DKO (n=4). Statistical analyses performed using Mann-Whitney tests; data represent mean ± SD.

(G) Alcian blue (cartilage) and alizarin red (bone) staining of the tympanic rings (white arrowheads) of P0 control (*Ehmt1$^{f/+}$;Ehmt2$^{f/+}$;Wnt1-Cre-*) and DKO pups, scale=50μm.

(H) Representative images of ribs from P0 control (*Ehmt1$^{f/f}$;Ehmt2$^{f/+}$;Wnt1-Cre-*) and DKO pups with alcian blue (cartilage) and alizarin red (bone) staining, scale=5mm.

(I) Representative images of tibia stained for alcian blue (cartilage) and alizarin red (bone), scale=500μm.

(J) Quantification of tibia lengths from P0 control (*Wnt1-Cre-*) and DKO pups. Statistical analyses performed using Mann-Whitney tests; ns: not significant; data represent mean ± SD.

(K) Table of PDA rates in P0 pups.

(L) Width of ductus arteriosus lumen (if present) in control and DKO P0 pups. LA: ligamentum arteriosum, DA: ductus arteriosus.

(M) Two additional control ($Ehmt1^{f/+}$;$Ehmt2^{f/f}$;*Wnt1-cre-*, Heart 1; $Ehmt1^{f/+}$;$Ehmt2^{f/+}$;*Wnt1-cre-*, Heart 2) and two DKO P0 hearts demonstrating a ligamentum arteriosum or ductus arteriosus. Ao: aorta; DA: ductus arteriosus; LA: ligamentum arteriosum; PA: pulmonary artery; PV: pulmonary valve, scale=500μm.

(N) Representative H&E coronal section images showing hearts of E17.5 control ($Ehmt1^{f/+}$;$Ehmt2^{f/+}$;*Wnt1-Cre-*) and DKO embryos. Ao: aorta; AoV: aortic valve; DA: ductus arteriosus; LV: left ventricle; PA: pulmonary artery; PV: pulmonary valve; RV: right ventricle; VSD: ventricular septal defect, scale=500μm.

S. Fig. 3

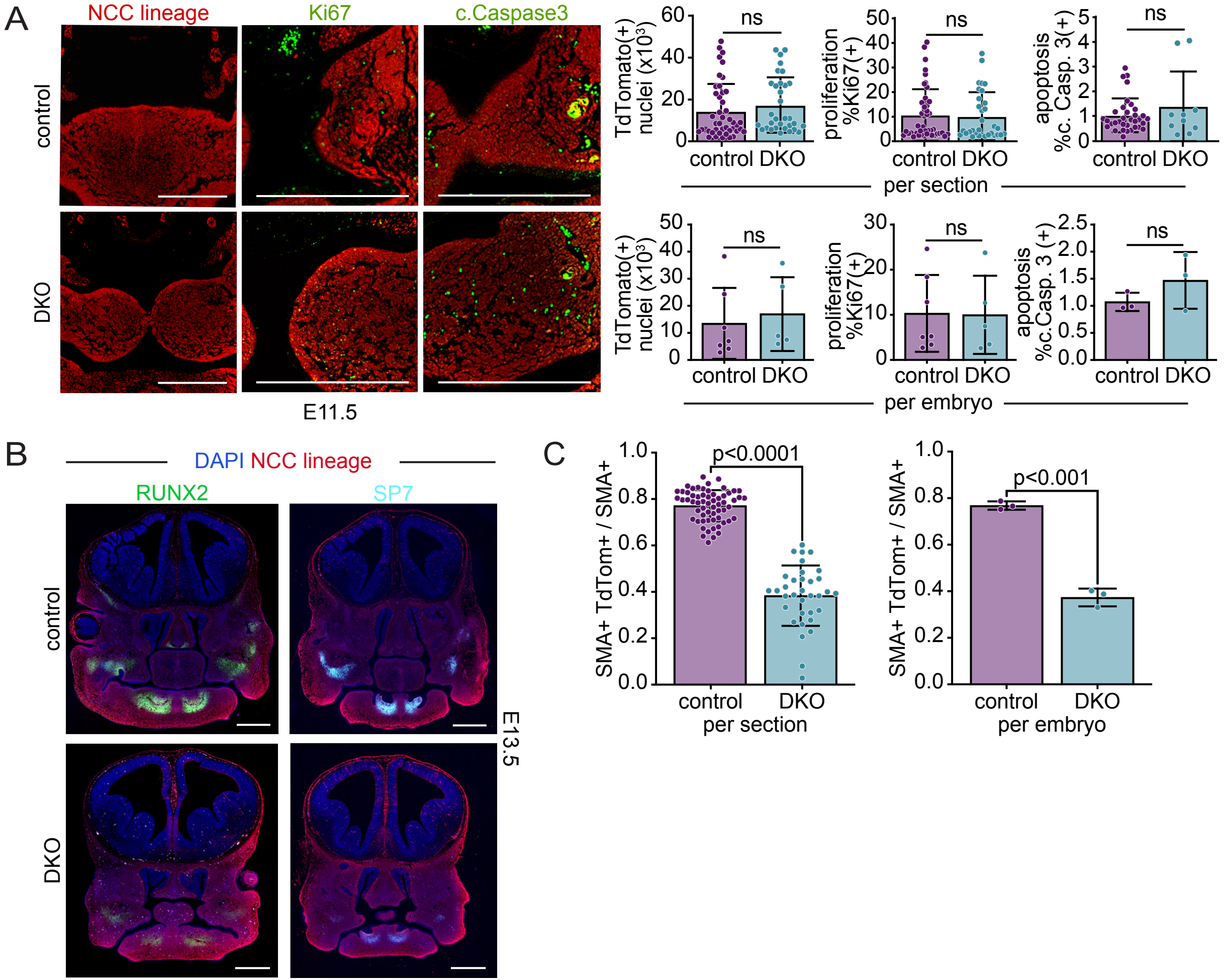

A
NCC lineage
Ki67
c.Caspase3
control
DKO
E11.5
TdTomato(+) nuclei (x10^3)
proliferation %Ki67(+)
apoptosis %c. Casp. 3(+)
ns
control DKO
per section
per embryo
B
DAPI NCC lineage
RUNX2
SP7
E13.5
C
SMA+ TdTom+ / SMA+
p<0.0001
p<0.001
control
DKO
per section
per embryo

Supplemental Figure 3

(A) Representative IF of branchial arches showing NCC lineage (red), Ki67 (green) or cleaved Caspase3 (green). Quantifications (right) of TdTomato+ nuclei and proliferation (Ki67+) or apoptosis (cleaved Caspase3+) as percentage of TdTomato+ cells. Analysis: Mann-Whitney t-tests; ns: not significant, data represent mean±SD. Each dot is one histological section (top row) or the average of all nuclei counted for one embryo (bottom row), n≥3 sections/embryo, 3 embryos/genotype, scale=500μm.

(B) Expanded panels (from D in main figure) of RUNX2 (green) and SP7 (cyan) with TdTomato (red) and DAPI (blue) IF in oral cavity, scale=500μm.

(C) Ratio of smooth muscle actin (SMA) cells surrounding the branchial arch arteries of E12.5 embryos that also express TdTomato. Each dot is a single histological section (left). Each dot is the average of all histological sections for one embryo (right). n =3 embryos/genotype. Statistical analyses performed using Mann-Whitney tests; data represent mean ± SD.

S. Fig. 4

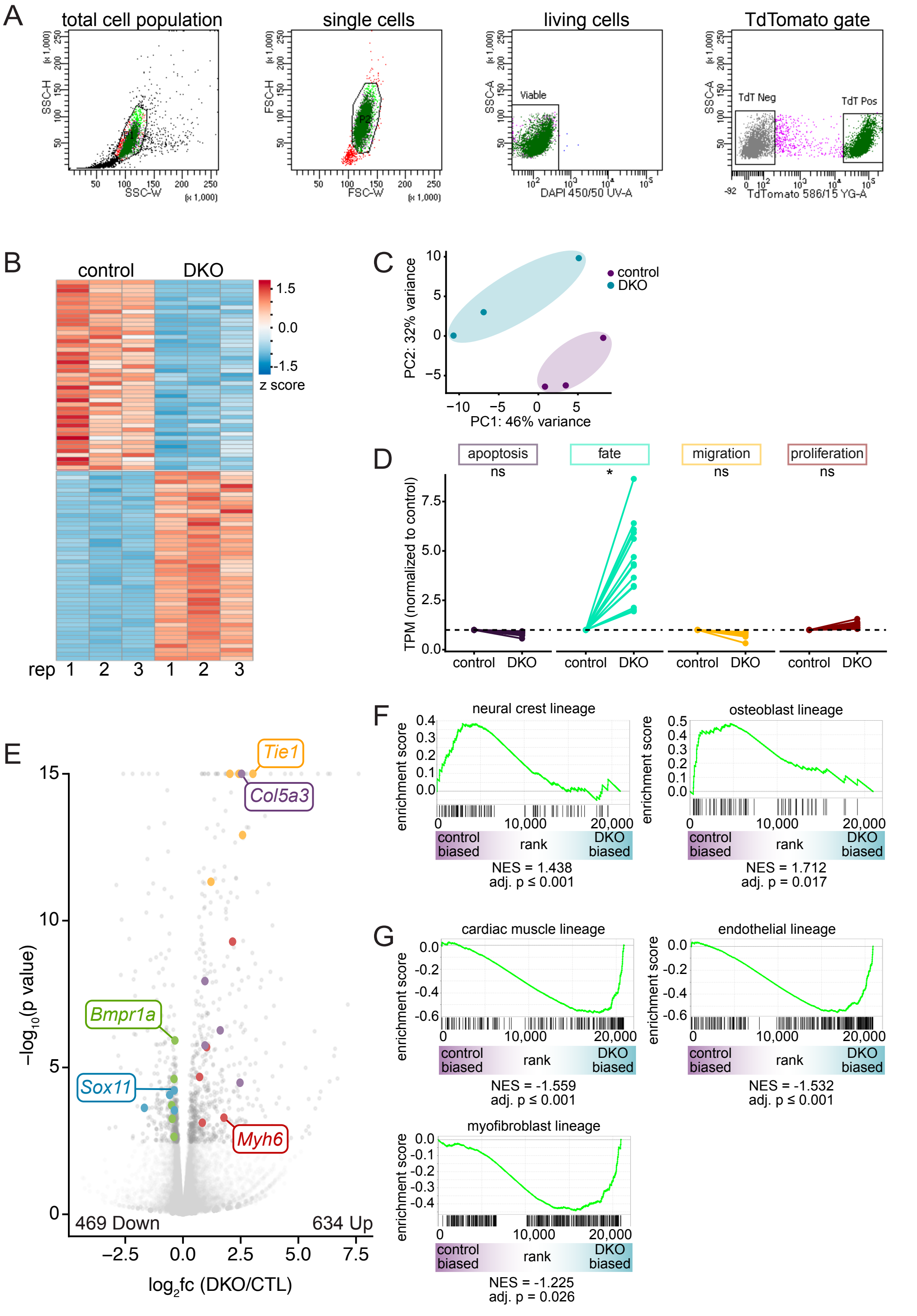
A
total cell population
SSC-H
SSC-W
P1
single cells
FSC-H
FSC-W
P2
living cells
SSC-A
Viable
DAPI 450/50 UV-A
TdTomato gate
SSC-A
TdT Neg
TdT Pos
TdTomato 586/15 YG-A
B
control
DKO
1.5
0.0
-1.5
z score
rep 1 2 3 1 2 3
C
control
DKO
PC2: 32% variance
PC1: 46% variance
D
apoptosis
ns
fate
*
migration
ns
proliferation
ns
TPM (normalized to control)
control DKO
E
Tie1
Col5a3
Bmpr1a
Sox11
Myh6
469 Down
634 Up
−log10(p value)
log2fc (DKO/CTL)
F
neural crest lineage
enrichment score
control biased
rank
DKO biased
NES = 1.438
adj. p ≤ 0.001
osteoblast lineage
NES = 1.712
adj. p = 0.017
G
cardiac muscle lineage
NES = -1.559
adj. p ≤ 0.001
endothelial lineage
NES = -1.532
adj. p ≤ 0.001
myofibroblast lineage
NES = -1.225
adj. p = 0.026

Supplemental Figure 4

(A) Flow cytometry gating strategy for neural crest cell (TdTomato+) isolation.

(B) Heatmap of top 45 down- and up-regulated differentially expressed genes (DEGs) with expression levels for each replicate sample isolated from TdTomato+ NCCs from branchial arches of E11.5 control (*Ehmt1*$^{+/+}$;*Ehmt2*$^{+/+}$;*Rosa*$^{TdTomato/+}$;*Wnt1-Cre*+) and DKO embryos.

(C) PCA plot of RNA-seq sample replicates.

(D) Line plot of fold change differences (TPM normalized to control) between E11.5 control (*Ehmt1*$^{+/+}$;*Ehmt2*$^{+/+}$;*Rosa*$^{TdTomato/+}$;*Wnt1-Cre*-) and DKO embryos for top 15 ranked genes from Gene Set Enrichment Analysis (GSEA) groups: apoptosis, cell fate, migration, and proliferation. Statistical analyses performed using Mann-Whitney tests; ns: not significant; *: p=0.04.

(E) RNA-seq from E11.5 branchial arch TdTomato+ NCCs shown as volcano plot of differentially expressed genes (DEGs) from DKO vs control. Illustrative genes from lineage-specific gene sets that were differentially enriched in control or DKO are indicated by color; blue: neural crest, green: osteoblast, red: cardiac muscle, orange: endothelial, purple: myofibroblast.

(F) GSEA plots of neural crest lineage and osteoblast lineage gene sets enriched in NCCs isolated from control (*Ehmt1*$^{+/+}$;*Ehmt2*$^{+/+}$;*Rosa*$^{TdTomato/+}$;*Wnt1-Cre*+) compared to DKO.

(G) GSEA plots of alternative fate-specific gene sets enriched in NCCs isolated from DKO compared to control (*Ehmt1*$^{+/+}$;*Ehmt2*$^{+/+}$;*Rosa*$^{TdTomato/+;}$*Wnt1-Cre*+).

Materials and Methods

Sex as a biological variable

All embryos at all stages were examined without regard to sex.

Animals

Ehmt1$^{tm1.1Tara}$ and *Ehmt1*$^{tm1.2Tara}$ mice were a generous gift from Kenneth Zaret, University of Pennsylvania, Philadelphia, PA; Alexander Tarakhovsky, Rockefeller University, New York, NY, and Anne Schaefer, Icahn School of Medicine, New York, NY. All mice, including *Wnt1-Cre* and *Rosa26*$^{TdTomato/TdTomato}$ mice, were maintained on a mixed background. In all cases of tissue collection, pregnant female mice were humanely euthanized prior to embryo retrieval. Pregnancies were timed by mating male and female mice overnight and, if a vaginal plug was observed, that day was counted as embryonic day 0.5 (E0.5). Postnatal day 0 (P0) pups were all collected > 6 hours after estimated time of birth. Control genotypes were as follows: *Ehmt1*$^{+/+}$;*Ehmt2*$^{+/+}$;*Rosa*$^{TdTomato/+}$;*Wnt1-Cre*+ (Figure1 A-B, D, F-I), *Ehmt1*$^{f/f}$;*Ehmt2*$^{f/+}$;*Wnt1-Cre*- (Figure1 C), *Ehmt1*$^{f/+}$;*Ehmt2*$^{f/f}$;*Wnt1-Cre*- (Figure1 E). Double knockout (“DKO”) genotype: *Ehmt1*$^{f/f}$;*Ehmt2*$^{f/f}$;*Rosa*$^{TdTomato/+}$;*Wnt1-Cre*+.

CUT&RUN

Branchial arches were isolated from E11.5 embryos, processed into a single cell suspension, and sorted on TdTomato signal as described below (see Flow Cytometry).

Following isolation, 500K TdTomato+ cells obtained from each embryo were used in CUT&RUN assays with the antibodies: H3K9me2 (Active Motif, cat# 39239), and Rabbit IgG (Cell Signaling Technologies, cat# 2729S). CUT&RUN was performed with CUTANA CUT&RUN Assays Kit (EpiCypher, cat#14-1048) per manufacturer's instructions and as previously reported [1].

Library prep and sequencing

DNA libraries for Next-Generation sequencing were prepared utilizing the KAPA HyperPrep Kit (Kapa Biosystems, cat# KK8502 07962347001) and NEBNext Multiplex Oligos for Illumina (New England Biolabs, cat# E6440S). To begin, samples were end repaired & A-tailed via incubation in a thermocycler for 30 min at 20°C followed by a 58°C incubation for 60 min. Next adapters were ligated per kit instructions with a 15-min incubation at 20°C with ligation buffer & DNA ligase, followed by a 37°C incubation for 15 min with NEB USER enzyme for an additional 15 min. Post-ligation cleanup was performed using 1.1x AMPure XP Beads (Beckman Coulter, cat# A63880). Next, the libraries were amplified per kit instructions with the following thermocycler conditions: 1 cycle of initial denaturation at 98°C for 45 s, 14 cycles of denaturation at 98°C for 15 s and annealing and extension at 60°C for 10 s, and 1 final extension cycle at 72°C for 1 min. Amplification was followed by an additional cleanup step with the 1.1x AMPure XP Beads and library quantification using the NEBNext Library Quantification Kit (cat# E7630) as per kit instructions. Library quality and fragment size was then determined via Agilent Technologies TapeStation using the High Sensitivity D1000 ScreenTape (cat# 5067–5584) and reagents (cat# 5067–5585). Libraries were then pooled and denatured via the

standard Illumina protocol. Finally, samples were paired-end sequenced on an Illumina NextSeq 1000.

Genome alignment and QC pipeline

For CUT&RUN samples, adapters were trimmed from fastq files using TrimGalore with default parameters (ver. 0.6.4 using Cutadapt 4.2 [2, 3]). The resulting reads were mapped with bowtie2 (ver. 2.5.0 [4]) with the parameters -X 2000 -N 1 –local –dovetail to the mm10 assembly. Only reads mapping to the 19 autosomal chromosomes were retained, and any reads mapping to the mm10 CUT&RUN blacklist [5] or the first 3 Mb of each chromosome (repetitive sequences near centromeres [6] were removed. Duplicates were marked using Picard (RRID:SCR_006525) Normalized bw files were produced for each H3K9me2 sample, normalizing against corresponding IgG sample from the corresponding embryo, using deeptools [7] bamCoverage with the parameters –operation log2 –extendReads –binSize 50. From these bw files, counts in tiled bins of 1 kb were calculated using deeptools multiBigwigSummary.

H3K9me2 Analysis

For each sample, z scores for each 1kb bin were calculated, then smoothed using a rolling mean incorporating the 5 windows flanking each side [8]. Regions with H3K9me2 smoothed z scores above the genome-wide median were assigned H3K9me2-modified.

Enrichment across annotated genomic features

Feature enrichment analysis was performed using Genomic Association Tester (GAT) [9]. The significance of overlap between sets of genomic intervals was calculated based on simulation using a permutation-based approach and accounting for genome organization

regions of low mapability. All enrichment analyses were subjected to 1000 simulations. The fold enrichment is expressed as the ratio of observed/expected (by chance). P values reflect an estimate of the probability to obtain an observed (or larger) overlap between two segment sets by chance and are reported as “significant” only if p <0.01.

Dissection and tissue processing

Embryos were dissected in cold PBS on ice, and all processing steps were conducted at 4°C unless otherwise noted.

Paraffin embedding and sectioning

Embryos were removed from their yolk sacs, fixed overnight in 4% PFA, rinsed 6 times in PBS and then dehydrated through an ethanol series. Embryos were then embedded in paraffin, sectioned to 6um, and mounted on slides.

Single cell isolation for immunofluorescence (IF) and RNA-seq

Tissues of interest (E11.5 branchial arches for RNA-seq, E13.5 oral cavity for IF) were separated from the remainder of the embryo via microdissection. Papain solution (Worthington, cat#LK003150) was added to the isolated tissue and then incubated at 37°C for 20-25 minutes with 600rpm agitation. Every 5-6 minutes, the samples were additionally mechanically disrupted by gentle pipetting to obtain a single cell suspension. The reaction was quenched according to manufacturer’s instructions. Cells were then centrifuged gently (300xg for 5 minutes), quenched papain solution removed, and cells washed twice with PBS.

Flow cytometry

Single cell suspensions isolated as described above were additionally filtered with a 40um cell strainer prior to flow cytometry. DAPI was used to identify non-viable cells. After gating for viability, cells were gated to identify singlets, and then on the presence or absence of TdTomato expression using filters for high purity (Supplementary Figure 4A). Sorting was conducted on an Accuri C6 flow cytometer or Symphony S6_A (both Becton, Dickinson and Company).

Gene set enrichment analysis (GSEA)

GSEA was performed via the GSEA 4.4.0 app build 18 [10, 11]. Gene sets were downloaded from the mouse Molecular Signatures Database (MSigDB), and included the following collections:

MH, hallmark gene sets:

HALLMARK_APOPTOSIS

M5, ontology gene sets:

GOBP_REGULATION_OF_CELL_POPULATION_PROLIFERATION

GOBP_CELL_MIGRATION

GOBP_NEURAL_CREST_CELL_DIFFERENTIATION

M8, cell type signature gene sets:

DESCARTES_ORGANOGENESIS_CARDIAC_MUSCLE_LINEAGES

DESCARTES_ORGANOGENESIS_ENDOTHELIAL_CELLS

ZHANG_UTERUS_C4_MYOFIBROBLAST

Genotyping

Genotyping was performed on embryonic yolk sacs and tissue and adult tail snips per standard genotyping protocols with the following primer sets.

Primer sequences

**Gene Target**	**Sequence 5' – 3'**
Ehmt1	Fwd: CTC AGT CAT TTA CTA AAG GTG C<br>Rev1: CCC GTG TAT TTG CAG TGC AAG<br>Rev2: TGC CTG GCA CAG AAG CCA TAG
Ehmt2	Fwd1: CAC GCT GCC TAG ATG GAG CAT GCC<br>Fwd2: CGA GGC TAT GAG AAT GTA CCC ATC CCC TG<br>Rev: GTG TGA GCC TGT GTT CTG GGG ATT A
Rosa26^{TdTomato}	Fwd: CAC TTG CTC TCC CAA AGT CG<br>Rev1: TAG TCT AAC TCG CGA CAC TG<br>Rev2: GTT ATG TAA CGC GGA ACT CC
Wnt1-Cre	Fwd: AAC CAC AGT CGT CAG AAC CG

	Rev: CCA GGT TAC GGA TAT AGT TCA TG

Histology and immunofluorescence

All microscopic images were collected on either a Leica DMi8S widefield microscope using a DFC7000 T camera or a Leica SP8 confocal.

Apoptosis staining

A minimum of 3 histological sections were stained and imaged per embryo, n=3 embryos/genotype. Similar regions were selected for analysis between animals using histological landmarks to ensure equal representation. Apoptosis was determined by positive cleaved Caspase3 staining as a percentage of total TdTomato+ cells (determined by DAPI & TdTomato staining). Cells were counted in a non-biased manner via Cell Profiler pipelines as described below.

Blocking peptide assay

Blocking peptide assay was performed as described previously and as demonstrated in Supplemental Figure 1F [12]. In brief, antibody for H3K9me1 (Active Motif, cat#39249, 1:1000) was first pre-incubated with either buffer (mock) or one of the following peptide versions of the first 20 residues (1-20aa) of the histone H3 tail: H3 unmodified (EpiCypher, cat# 12-0001), mono-methylated Lys9 (H3K9me1, EpiCypher, cat# 12-0012), di-methylated Lys9 (H3K9me2, EpiCypher, cat# 12-0010), and tri-methylated Lys9 (H3K9me3, EpiCypher, cat# 12-0012). The antibody was incubated with the selected peptide (1:250) for 1 hour at room temperature then used in standard immunofluorescent

protocol. Murine C2C12 cells were then stained with the pre-blocked antibody/peptide mixtures. Confocal images were taken with the same settings across conditions.

Bone staining

Bone staining was performed on 6 litters (n ≥4 pups per genotype) of P0 pups via standard protocol, ([13], protocol 3.2), with a few notable exceptions. KOH preclearing of tissues after alcian blue staining (step 7) was conducted overnight at 4°C. Additionally, alizarin red staining was conducted overnight at 4°C (step 8). Finally, stained P0 skeletons were then cleared in a 80:20 1%KOH:glycerol solution. Skeletons were kept in this solution until the residual tissue was cleared (24-48 hours), changing the solution every 24 hours. Samples were then stored in 100% glycerol for long term storage and imaging.

Cardiac Morphometrics

There were a total of 9 controls and 3 DKOs examined at E17.5, and 46 controls and 8 DKOs at P0.

Cell profiler

Images to be analyzed were edited to only include the region of interest (i.e., branchial arches) prior to analysis. A pipeline was then created in Cell Profiler ([14], https://sites.broadinstitute.org/cellprofiler/) to allow for non-biased cell counting as follows. First, microscope images were converted to binary channels. Primary objects (DAPI stained nuclei) were then identified, constituting "total cells", the denominator for quantification. If required, secondary objects (nuclei stain of interest, i.e. Ki67, cleaved Casp3, TdTomato) were identified. Finally, primary and secondary objects were overlayed to allow for pruning of any background staining, and the final counts were recorded and

exported into .csv files for further compilation. The authors were blinded to genotypes until after counts were processed and final totals tabulated. All analyses included a minimum of 3 sections/embryo and 3 embryos/genotype.

Hematoxylin and eosin (H&E), Masson trichrome, and immunohistochemistry (IHC)

H&E and Masson's trichrome stains were performed according to a standard protocol. For IHC, after rehydration, antigen retrieval was performed with either citrate buffer or EDTA pH9.0. Sections were then incubated overnight with primary antibody at 4°C, followed by secondary antibody for 1 hour at room temperature. Finally slides were briefly incubated with DAPI and mounted with Vectashield (Vector Labs H-1000).

Primary antibodies and concentrations for IHC

Antibody Target	Catalog Number	Company	Dilution
Cleaved Caspase 3	9664	Cell Signaling	1:50
G9a	A8620A	Invitrogen	1:100
GLP	PP-B0422-00	R&D Systems	1:200
H3K9me1	39249	Active Motif	1:200
H3K9me2	39239	Active Motif	1:200
H3K9me3	AB8898	Abcam	1:200
Ki67	AB16667	Abcam	1:50
RUNX2	SC10758	Santa Cruz	1:100

SMA	A2547	Sigma	1:400
SP7	AB209484	Abcam	1:100
TdTomato	ABIN6254170	Antibodies Online	1:100

Proliferation assay

A minimum of 3 histological sections were stained and imaged per embryo, n=3 embryos/genotype. Similar regions were selected for analysis between animals using histological landmarks to ensure accurate representation. Proliferation was determined by positive Ki67 staining as a percentage of total TdTomato+ cells (determined by DAPI and TdTomato staining). Cells were counted in a non-biased manner via Cell Profiler pipelines as described above.

TdTomato(+) nuclei analysis

TdTomato(+) cell counts were derived by reporting the total number of TdTomato(+) cells from both the apoptosis and proliferation assays (n =6 embryos/genotype, >=3 sections/embryo).

RNAscope

RNAscope was performed via the Multiplex Fluorescent Reagent Kit v2 User Manual (323100-USM Date 04032917, Advanced Cell Diagnostics, Inc). In brief, FPFE tissue sections were deparaffinized and pretreated to permeabilize the tissue. Next, slides were hybridized with transcript-specific probes and the signal amplified.

Probes: Mm-Ehmt1-C1 (Advanced Cell Diagnostics, Inc, cat#816281) and Mm-Ehmt2-C2 (Advanced Cell Diagnostics, Inc, cat# 547151).

RNA extraction

RNA was extracted via Qiagen RNeasy Plus Mini Kit (cat#74134) per manufacturer's instructions. Isolated and sorted cells (see above) were stored in RNAlater at -20°C.

RNA library preparation and sequencing

Extracted RNA was converted to cDNA and libraries were prepared via the Illumina Stranded mRNA Prep (cat#20040532) per manufacturer’s instructions. Samples were sequenced on a Nextseq 1000 with 2x 40 base pairs and 80 total cycles. (P2 reagent cartridge, cat#2004468, Illumina).

Sequencing alignment, QC pipeline & DEG analysis

Adapters were trimmed from fastq files using TrimGalore [3] with default parameters. Reads were mapped to the mm10 genome using STAR version 2.7.10a [15] with the parameters *--peOverlapNbasesMin 5 --alignIntronMax 100000 --outFilterScoreMinOverLread 0.9 --outFilterMatchNminOverLread 0.9 --outFilterMismatchNoverLmax 0.05 --outFilterMatchNmin 16*. Duplicates were marked by Picard (https://broadinstitute.github.io/picard/) and removed from further analyses. Read counts on genes were calculated using an in-house R script utilizing the GenomicRanges

[16] functions *summarizeOverlaps*. Differentially expressed genes were identified using DESeq2 [17].

Data availability

All data needed to evaluate the conclusions in this paper are presented in the methods, manuscript or have been deposited at NCBI Gene Expression Omnibus (GEO; www.ncbi.nlm.nih.gov/geo/) and available under accession number GSE334888. All reported data values are available in the Supporting Data file.

Statistics

For all statistics, p values less than 0.05 were considered significant.

Study approval

All experiments using mice were approved by the Institutional Animal Care and Use Committee (IACUC) of the University of Pennsylvania, and mice were housed in facilities accredited by the American Association for Accreditation of Laboratory Animal Care (AAALAC).

Acknowledgments

We thank Anna Kiseleva for assistance with flow cytometry as well as the Penn Cytomics and Cell Sorting Shared Resource Laboratory at the University of Pennsylvania (RRID:SCR_022376). We also thank all members of the Epstein and Jain labs for their ideas and support.

Author Contributions

DBV, AP, CLS, RJ, and JAE conceived of the study, designed experiments, interpreted data, and wrote the manuscript. DBV, AP, LL, JL, LW, and MC collected and analyzed data. DBV, EJS, and CLS performed formal analyses of CUT&RUN and RNA-seq data. CLS, RJ, and JAE supervised the project. DBV, AP, RJ, and JAE acquired study funding. All authors contributed to review and editing of the final manuscript.

REFERENCES

1. Fulmer, D., et al., *Reorganization of H3K9me2-modified chromatin regions during mouse embryonic development.* Dev Biol, 2025. **528**: p. 188–203.
2. Kechin, A., et al., *cutPrimers: A New Tool for Accurate Cutting of Primers from Reads of Targeted Next Generation Sequencing.* J Comput Biol, 2017. **24**(11): p. 1138–1143.
3. Krueger, F., *FelixKrueger/TrimGalore: v0.6.10 - add default decompression path (0.6.10)*, F. James, Editor. 2023, Zenodo.
4. Langmead, B. and S.L. Salzberg, *Fast gapped-read alignment with Bowtie 2.* Nat Methods, 2012. **9**(4): p. 357–9.
5. Nordin, A., et al., *The CUT&RUN suspect list of problematic regions of the genome.* Genome Biol, 2023. **24**(1): p. 185.
6. Cox, A., et al., *A new standard genetic map for the laboratory mouse.* Genetics, 2009. **182**(4): p. 1335–44.
7. Ramirez, F., et al., *deepTools2: a next generation web server for deep-sequencing data analysis.* Nucleic Acids Res, 2016. **44**(W1): p. W160–5.
8. Zeileis A, G.G., *Zoo: S3 infrastructure for regular and irregular time series.* J. Stat. Software, 2005. **14**(6): p. 1–27.
9. Heger, A., et al., *GAT: a simulation framework for testing the association of genomic intervals.* Bioinformatics, 2013. **29**(16): p. 2046–8.

10. Mootha, V.K., et al., *PGC-1alpha-responsive genes involved in oxidative phosphorylation are coordinately downregulated in human diabetes.* Nat Genet, 2003. **34**(3): p. 267–73.

11. Subramanian, A., et al., *Gene set enrichment analysis: a knowledge-based approach for interpreting genome-wide expression profiles.* Proc Natl Acad Sci U S A, 2005. **102**(43): p. 15545–50.

12. Poleshko, A., et al., *H3K9me2 orchestrates inheritance of spatial positioning of peripheral heterochromatin through mitosis.* Elife, 2019. **8**.

13. Rigueur, D. and K.M. Lyons, *Whole-mount skeletal staining.* Methods Mol Biol, 2014. **1130**: p. 113–121.

14. Stirling, D.R., et al., *CellProfiler 4: improvements in speed, utility and usability.* BMC Bioinformatics, 2021. **22**(1): p. 433.

15. Dobin, A., et al., *STAR: ultrafast universal RNA-seq aligner.* Bioinformatics, 2013. **29**(1): p. 15–21.

16. Lawrence, M., et al., *Software for computing and annotating genomic ranges.* PLoS Comput Biol, 2013. **9**(8): p. e1003118.

17. Love, M.I., W. Huber, and S. Anders, *Moderated estimation of fold change and dispersion for RNA-seq data with DESeq2.* Genome Biol, 2014. **15**(12): p. 550.
